# Cultural Sources of Strength and Resilience: A Case Study of Holistic Wellness Boxes for COVID-19 Response in Indigenous Communities

**DOI:** 10.3389/fsoc.2021.612637

**Published:** 2021-02-22

**Authors:** Kevalin M. W. Aulandez, Melissa L. Walls, Nicole M. Weiss, Kelley J. Sittner, Stefanie L. Gillson, Elizabeth N. Tennessen, Tara L. Maudrie, Ailee M. Leppi, Emma J. Rothwell, Athena R. Bolton-Steiner, Miigis B. Gonzalez

**Affiliations:** ^1^ Johns Hopkins Center for American Indian Health, International Health Department, Johns Hopkins Bloomberg School of Public Health, Duluth, MN, United States; ^2^ Department of Sociology, Oklahoma State University, Stillwater, OK, United States; ^3^ Department of Psychiatry, Yale University School of Medicine, New Haven, CT, United States

**Keywords:** Indigenous peoples, community-based participatory research, relief work, pandemics, COVID-19, coronavirus, holistic health

## Abstract

The COVID-19 pandemic has had disproportionately severe impacts on Indigenous peoples in the United States compared to non-Indigenous populations. In addition to the threat of viral infection, COVID-19 poses increased risk for psychosocial stress that may widen already existing physical, mental, and behavioral health inequities experienced by Indigenous communities. In recognition of the impact of COVID-19 related psychosocial stressors on our tribal community partners, the Johns Hopkins Center for American Indian Health Great Lakes Hub began sending holistic wellness boxes to our community partners in 11 tribal communities in the Midwestern United States and Canada in summer of 2020. Designed specifically to draw on culturally relevant sources of strength and resilience, these boxes contained a variety of items to support mental, emotional, cultural, and physical wellbeing. Feedback from recipients suggest that these wellness boxes provided a unique form of COVID-19 relief. Additional Johns Hopkins Center for American Indian Health offices have begun to adapt wellness boxes for the cultural context of their regions. This case study describes the conceptualization, creation, and contents of these wellness boxes and orients this intervention within a reflection on foundations of community-based participatory research, holistic relief, and drawing on cultural strengths in responding to COVID-19.

## Introduction

American Indian and Alaska Native peoples, hereafter referred to as Indigenous peoples, have long experienced poorer mental and physical health outcomes than non-Indigenous Americans, including high rates of suicide, type 2 diabetes, cardiovascular disease, liver disease, and a variety of infectious diseases (Indian Health Service, 2019). From the biological warfare of smallpox brought by colonizers to Indigenous peoples in the 1790s to the modern day COVID-19 pandemic, infectious diseases continue to be a significant and disproportionate cause of morbidity and mortality in Indigenous communities (Indian Health Service, 2019; Hatcher et al., 2020). Health inequities are rooted in social determinants of health such as colonization, systemic racism, higher rates of poverty, and unequal access to education, and these health inequities contribute to higher incidence rates of COVID-19 among Indigenous people in the United States (King et al., 2009; Walls and Whitbeck, 2012; Bombay et al., 2014). Exclusion of Indigenous communities in public health surveillance and inaccuracies in existing data mean that the full impacts of COVID-19 in these communities are unknown (U.S. Commission on Civil Rights, 2018). Even with these limitations in mind, existing data reveal that Indigenous communities are experiencing the highest documented rates of COVID-19 disease and death in the United States (Indian Health Service, 2020).

COVID-19 in Indigenous communities also elevates psychosocial stressors and exacerbates health inequities. Psychosocial stress is an etiological contributor to behavioral, mental, and physical health problems (Pearlin and Bierman, 2013). Prior research demonstrates that marginalized groups experience more frequent and severe psychosocial stressors (Turner et al., 1995), and the concentration and accumulation of stress leads to poorer health outcomes and impedes treatment for existing health issues (Dedert et al., 2009; DiMaggio et al., 2009). Historical and contemporary inequities result in greater burdens of stress for Indigenous peoples (Tiedt and Brown, 2014). A recent survey of Indigenous peoples in Canada found that six in 10 participants reported worse mental health since pandemic restrictions began; worsening mental health related to COVID-19 was reported at disproportionate rates among Indigenous compared to non-Indigenous participants (Canada Statistics, 2020).

## Context

The Johns Hopkins Center for American Indian Health (CAIH) partners with Indigenous communities across North America to promote health, health leadership, and self-sufficiency. Founded in 1991, the CAIH works collaboratively with tribal partners in the areas of infectious disease prevention and treatment, behavioral health promotion, and training of Indigenous health professionals. In August of 2019, the authorship team of the current manuscript joined the CAIH as the CAIH Great Lakes Hub (GLH). The GLH research team includes over 100 tribal members across 11 Indigenous communities in the upper Midwestern United States and Canada. Our team is engaged in numerous health-related studies and intervention projects, primarily on reservation/reserve lands. All GLH studies and projects are co-led by university-based investigators and Community Research Councils comprised of tribal community members. Many of our team members have worked in partnership for nearly 20 years. All of our projects are rooted in community-based participatory research (CBPR), an approach to research that recognizes communities as necessary and equal partners in the creation of knowledge and in its application (Israel et al., 1998). The approaches of CBPR have been compared to those of social movement paradigms due to a number of shared characteristics (Tremblay et al., 2017; Tremblay et al., 2018). Underpinning social movements is collective framing of, and actions to remedy, the underlying causes of societal ills that lead to poor outcomes among particular populations (Masters and Osborn, 2010). Such shared framing and action can be developed and mobilized through CBPR (Tremblay et al., 2018; Masters and Osborn, 2010) and has enabled our team to work together on a variety of relief efforts during the COVID-19 pandemic. In this manuscript, we explore a case study of the conception, creation, and contents of holistic wellness boxes for psychosocial support during COVID-19 and place this intervention within the context of nearly two decades of CBPR practice.

As the COVID-19 pandemic progressed, our research team temporarily paused projects, regrouped, and adapted to web and phone-based modalities. Tribally-based team members persisted in their work even as communities closed key operations and schools, caregiving roles were amplified, and financial, health, and race-related stressors peaked. Yet, the strains of social isolation and accumulating pressures were palpable among our team, particularly as the pandemic contributed to disconnection from seasonal cultural activities, ceremonies, and social gatherings—all known protective factors for health in Indigenous communities (Oré et al., 2016).

## Description of Key Elements

In March of 2020, the CAIH moved quickly to dedicate resources to respond to the COVID-19 pandemic. Deep seated relationships, existing team structures, and momentum of our CBPR team enabled us to shift efforts rapidly to address the pandemic. One response effort by the GLH was the creation of holistic wellness boxes, which is the focus of the current manuscript. In April of 2020, both U.S. states within which our research team operates were granted disaster declarations by the federal government (The Office of the Governor, State of Wisconsin, 2020; The White House, 2020). In recognizing the potential value of literature related to disasters and emergencies to the local reality of COVID-19, we used existing guidance for disaster response, and feedback from community partners, to guide initial relief efforts (Sphere, 2018). Our team compiled example response approaches into a brief, online needs assessment that was distributed to existing community partners. The needs assessment asked community partners to identify the top three most pressing needs in their communities related to the COVID-19 pandemic and which potential relief approaches could best meet these needs. We considered several factors to decide which COVID-19 response activities were included in the needs assessment. These factors included: 1) relevance to the local reality of the COVID-19 pandemic; 2) support in existing literature on disaster response; 3) feasibility within the resources and capacity of the CAIH GLH and; 4) compatibility with strengths of existing community partnerships. Additionally, community partners were invited to participate in regular video calls to discuss community needs. These calls were held two times per month from April to June 2020. Feedback from community partners led us to focus initial COVID-19 response efforts on the distribution of food, household goods, and personal protective equipment in partnership with tribal agencies and clinics.

As the pandemic persisted and our team continued regular video calls, community-based research team members voiced an increasing need for a more holistic COVID-19 response approach that would address not only physical health, but also cultural, spiritual, emotional, and mental health. Years of collaborative work on issues of Indigenous health equity allowed our team to draw on a shared understanding of culture as a source of strength and healing that can strengthen efforts to address modern day health inequities—including the disproportionate impact of COVID-19 on Indigenous peoples. Thus, to meet the need for culturally relevant psychosocial support, we designed and distributed holistic wellness boxes and aimed for box contents to be: 1) evidence-based; 2) responsive to the needs voiced by community partners; 3) culturally relevant and; 4) logistically feasible. We also gave attention to sourcing box supplies from local and Indigenous retailers as much as possible.

### Holistic Wellness Box Contents

#### Gifts to Bring Calm, Relieve Stress, and Strengthen Cultural Connection

Supplementary Table 1 includes a full list of holistic box contents. Traditional cultural and spiritual practices have been shown to be protective for mental and physical health among Indigenous populations (Brockie et al., 2018). Thus, strengthening cultural connection during the time of COVID-19 is one approach to building mental and emotional resilience. To foster cultural connection, the boxes included sage to be used for smudging—a traditional practice of many Indigenous communities. The boxes also included cards with teachings shared by Ojibwe Elder and cultural advisor, Lee “Obizaan” Staples, (who gave permission for the teachings to be recorded and printed) on topics such as dealing with stress, taking care of oneself, and dealing with challenging emotions. Elders play an important role in many tribal communities as stewards of cultural knowledge and history and the cross-generational sharing of knowledge fosters resilience and promotes health (Cwik et al., 2019). The boxes also included ribbons featuring Ojibwe floral designs for use in making traditional crafts and resources for practicing mindfulness. Mindfulness practices have been shown to correlate with lower stress levels, “better self-regulation, less mind wandering and decreased suicidal thoughts” (Thao and Gobert, 2013, p. 12). As an aid for mindfulness and relaxation, the boxes also included lavender essential oil, which a number of studies have found to lessen anxiety symptoms and improve sleep quality (Conrad and Adams, 2012; Karadag et al., 2015).

#### Gifts to Nourish Body and Spirit

Wild rice is a culturally, spiritually, and historically significant food for Ojibwe communities. Several studies have confirmed that a diet based in traditional foods is protective against diet-related diseases among Indigenous populations (McLaughlin, 2010; Satterfield et al., 2016). Traditional foods are not only protective for physical health, however. During the 2018 Native Nutrition Conference, a participant shared the following insight.

*Traditional Indigenous foods are highly nutritious. They’re healing, spiritual and comforting. They are home to our people. When we provide traditional foods to our people, we are healing as well.* (Terra Somma and Ladywithafan Designs, 2018, p. 3)

Informed by these understandings, our team included wild rice and recipes using traditional foods in the wellness boxes. Our goal was to promote nutritional health in a time when many are facing increased risk of food insecurity due to COVID-19 induced economic vulnerability, and to strengthen connection to culture as a means to support mental and emotional wellbeing.

#### Gifts to Support the Mental and Physical Health of Children

To support the social and emotional wellbeing of the youngest members of our partner communities, the boxes included educational and engaging story and activity books for children, many of whom were schooling from home for the first time and experiencing heightened stress due to the pandemic. Of particular note was the inclusion of the story book published by the CAIH, *Our Smallest Warriors, Our Strongest Medicine: Overcoming COVID-19* (Allison-Burbank et al., 2020; Johns Hopkins Center for American Indian Health, 2020). This story book utilizes Indigenous storytelling while providing public health education and cultural strengths-based messages for children in the kindergarten to fifth grade age group. The story provides a hopeful and empowering narrative to promote the mental health and resilience of Indigenous children and families coping with the COVID-19 pandemic. This book was culturally adapted from the story *My Hero is You: How Kids Can Fight COVID-19*! developed by the Inter-Agency Standing Committee Reference Group on Mental Health and Psychosocial Support (Inter-Agency Standing Committee, 2020; Johns Hopkins Center for American Indian Health, 2020). The adaption process was led by a team of Indigenous and non-Indigenous allied experts in child development, mental health, and health communication as well as public health professionals and an Indigenous youth artist who created the book illustrations (O’Keefe et al., 2020). This book is freely available online alongside downloadable coloring pages, children’s activities, and parent resources.

The boxes also included *Minwanjige Mino-bimaadizi* activity books, published by the University of Minnesota Medical School, which integrate Ojibwe language and culture to educate children about healthy eating (Dietz-Castel, 2020; Kosobuski et al., 2020). Also included in the boxes were crayons for children to use to complete activities and coloring pages.

#### Resources to Prevent the Spread of COVID-19

To prevent transmission of the COVID-19 virus, the boxes included cloth face masks and hand soap and/or bottles of hand sanitizer. We included these items to encourage healthy behaviors such as hand washing and wearing face coverings, behaviors that are in line with current recommendations to slow the spread of the COVID-19 coronavirus (National Center for Immunization and Respiratory Diseases, 2020).

### Holistic Wellness Box Construction and Distribution

The holistic wellness boxes were prepared by CAIH GLH team members in our office in Duluth, MN. Our team distributed roughly 110 holistic wellness boxes by mail to community-based research team members and other community partners. Mailing of the boxes happened over the course of several weeks and each box was tracked to ensure it was delivered. Our team sent the boxes in batches by community, so that all recipients within a specific community would receive their boxes at roughly the same time. When each batch of boxes was mailed, the CAIH GLH Study Coordinator sent an email to box recipients letting them know to look for the arrival of a package. Additional CAIH offices in the southwestern United States are currently adapting the holistic wellness boxes for distribution in their region.

## Discussion

Response from wellness box recipients was incredibly positive. Several themes emerged in the responses to the wellness boxes by recipients. One such theme was that the boxes provided a much needed source of upliftment, with one recipient sharing that receiving the box, “lit up my world.” Another theme that emerged was appreciation for culturally specific items. Several recipients, in voicing thanks for receiving their box, specifically mentioned the culturally significant elements of the boxes. One participant shared that, in response to receiving a wellness box, they would heed our message to “focus on self-care.” In the following sections of this manuscript, we will reflect on three areas that may have contributed to the boxes receiving such positive response: 1) a foundation of CBPR; 2) a holistic approach to relief and; 3) fostering resilience and wellbeing by drawing on cultural strengths.

### Building on a Foundation of Community-Based Participatory Research

Fundamental to our COVID-19 response approach and the creation of the wellness boxes was communication and engagement with community partners built on a foundation of many years of partnership and collaboration. As mentioned above, all of the work of our team operates within a framework of CBPR. CBPR involves equal partnership between communities and academic institutions in the generation and application of knowledge (Israel et al., 1998). Defining characteristics of work rooted in CBPR include an iterative approach, multi-directional co-learning, and a strengths-based process that recognizes the unique contributions of all parties (Israel et al., 1998). Both a necessity and biproduct of longstanding CBPR partnerships with tribal communities is a foundation of respect and trust (Elm and Handeland, 2020). During COVID-19 response efforts, our team was able to draw on this foundation, as well as on nearly two decades of CBPR which has yielded considerable development in the key components of social movements as outlined by Masters and Osborn (2010). **Building a base and alliances**. Our research team includes over 100 individuals from 11 tribal nations, many of whom are members of tribal government or who represent tribal agencies or clinics. **Leadership**. Each partner community is represented in our CBPR efforts by a Community Research Council, the members of whom share leadership with university based researchers. **Ideas and vision**. As already discussed, many years of partnership in working toward Indigenous health equity has enabled our team to reach a unified understanding of historically rooted social determinants as the underlying cause of modern day health inequities and of the capacity of culture to promote health. **Infrastructure for advocacy**. Through networks and relationships forged over decades with individuals, organizations, and tribal governments, the research of our team has led to the implementation of a number of evidence-based, culturally relevant interventions in partnership with tribal communities. During COVID-19, established patterns of communication and collaboration with community partners allowed us to understand what needs were most urgent, quickly adapt to changing priorities, tailor the wellness boxes to address contextually specific stressors, and draw on community specific strengths.

### Holistic Relief

The COVID-19 pandemic is disproportionately affecting the physical and psychosocial health of Indigenous communities (Canada Statistic, 2020; Indian Health Service, 2020). Disasters often have an inequitable impact on the mental health of historically marginalized populations as new disaster-induced stressors are layered on existing social, political, and economic disenfranchisement (Marsella and Christopher, 2004). Thus, it is paramount that COVID-19 response in Indigenous communities address health holistically with attention to physical, spiritual, emotional, cultural, and mental factors. The National Voluntary Organizations Active in Disaster recommends that, among many other components, spiritual care and promotion of self-care be integrated into early psychological interventions in disaster settings (Everly et al., 2008). Similarly, fostering appropriate cultural and spiritual healing practices is encouraged by the Inter-Agency Standing Committee Reference Group for Mental Health and Psychosocial Support in Emergency Settings to promote psychosocial wellbeing (Inter-Agency Standing Committee Reference Group for Mental and Psychosocial Support in Emergency Settings, 2010). Through integration of culturally relevant supplies to promote physical and psychosocial health, our team strove to support holistic wellbeing and draw on existing sources of resilience already embedded within Indigenous individuals and communities.

### Fostering Resilience and Wellbeing Through Drawing on Cultural Strengths

A foundational element of CBPR is building on the strengths that already exist within communities (Israel et al., 1998). Similarly, fostering “natural recovery mechanisms” is recommended in early mental health interventions following disaster events (National Institute of Mental Health, 2002; Everly et al., 2008, p. 409). Cultural connection and practices have been shown to be protective for physical and psychosocial health in Indigenous communities (Brockie et al., 2018). Indigenous peoples believe wholeheartedly and unquestionably in the healing power of culture (Hartmann and Gone, 2012; Gone, 2013; Moghaddam et al., 2015). This is further evidenced by the fact that health interventions that include cultural components have been shown to be more effective and generate greater community approval from Indigenous populations (Lowe, 2006; Walls et al., 2006; Hartmann and Gone, 2012; Goodkind et al., 2015). Involvement in traditional and spiritual activities (Kading et al., 2015; Bear et al., 2018) and connection to community (Greenfield and Marks, 2010) have been associated with positive mental health. Indigenous language use has been associated with lower youth suicide (Hallett et al., 2007) and lower rates of diabetes (Oster et al., 2014), and connection to land and nature has been linked to individual and community healing and resilience (Ritchie et al., 2014; Goodkind et al., 2015; Schultz et al., 2016). Culture is so vital to Indigenous health that accepted models of health and health care for this population rely on a foundation of culture (Lowe and Struthers, 2001; Hill, 2006; Ullrich, 2019).

Indigenous communities hold within themselves reservoirs of cultural knowledge, teachings, and practices that have the capacity to protect and promote holistic health. The boxes provided by the CAIH GLH are one example—rooted in cultural strengths and scientific evidence—of an intervention designed and driven by community feedback to holistically address wellbeing in the time of COVID-19.

## Limitations

Due to the need to distribute boxes quickly, there was no empirical evaluation of the impact of the holistic boxes on health outcomes. Developing a pre/post evaluation would have considerably delayed distribution of the boxes. Thus, the impact of the wellness boxes on COVID-19 related physical and psychosocial health outcomes cannot be fully known. Future similar interventions could investigate the empirical impact of similar, culturally and scientifically grounded care packages on physical and psychosocial health. There are numerous innovative and Indigenous approaches to evaluation including storytelling, sharing circles, photovoice, and open-ended interviews, which may afford appropriate venues through which to explore the impact of these forms of gifting on holistic health (Kovach, 2009; Bennet et al., 2019). Despite our lack of evaluation, anecdotal feedback suggests that the holistic boxes facilitated relational connections and a sense of belonging, each of which are critical components of wellbeing (Hill, 2006; Ullrich, 2019).

## Conclusion

For centuries, the Indigenous populations of North America have experienced disproportionate impacts from infectious diseases (Indian Health Service, 2019; Hatcher et al., 2020). The emergence of the COVID-19 pandemic has layered new psychosocial and physical health risks on existing health inequities in Indigenous communities (Indian Health Service, 2019; Canada Statistics, 2020). In seeking to provide COVID-19 relief in Indigenous communities, the voices of those most affected should be considered the central authority on their own needs and systems for frequent, bi-directional communication based on mutual respect and trust should be established in order to ensure responsive action and honor the knowledge and needs of community members.

COVID-19 has taken a toll not only on physical health, but also on mental, spiritual, emotional, and cultural health. A holistic response approach that strengthens existing sources of strength, resilience, and healing may be a useful addition to interventions striving to address physical and psychosocial wellbeing during COVID-19. In Indigenous communities, drawing on cultural strengths, knowledge, and teachings can be a powerful tool for protecting and promoting health (Lowe, 2006; Walls et al., 2006; Hartmann and Gone, 2012; Goodkind et al., 2015). The holistic wellness boxes prepared and distributed by our team are an example of such an intervention that, based both in scientific evidence and cultural knowledge and grounded in years of CBPR, provided a unique form of relief during the COVID-19 pandemic.

## Data Availability

The original contributions presented in the study are included in the article/Supplementary Material Further inquiries can be directed to the corresponding author.
