# Supplementary material for: Cultural Sources of Strength and Resilience: A Case Study of Holistic Wellness Boxes for COVID-19 Response in Indigenous Communities
Source: Front Sociol. 2021 Feb 22;6:612637. doi: 10.3389/fsoc.2021.612637 (PMC8022692; doi:10.3389/fsoc.2021.612637)
Supplement: Supplementary file 1 [file Table1.pdf]

Supplementary Material

Type of Resource	Content of Holistic Wellness Boxes
Resources to bring calm, relieve stress, and strengthen cultural connection	Sage for smudging
	Ribbon featuring Ojibwe floral designs
	Lavender essential oil
	Ojibwe Elder's teachings about dealing with anxiety and stress
	Mental health resource list
	Mindfulness exercises
Resources to nourish body and spirit	Wild rice
	Recipes using traditional foods
Resources to support the mental and physical health of children	Our Smallest Warriors, Our Strongest Medicine: Overcoming COVID-19 children's book and coloring pages (Allison-Burbank et al., 2020)
	Minwanjige, Mino-bimaadizi (Eat Well, Live Well) activity books about healthy eating (Kosobuski et al., 2020)
	Crayons
Resources to prevent the spread of COVID-19	Cloth mask
	Bar soap and/or hand sanitizer

Supplementary Table 1. Contents of the holistic wellness boxes distributed by the Center for American Indian Health Great Lakes Hub

References

Allison-Burbank, J., Haroz, E. E., Ingalls, A., Kee, C., Martin, L., Masten, K, et al. (2020). *Our smallest warriors, our strongest medicine: overcoming COVID-19*. Baltimore, MD: The Johns Hopkins Center for American Indian Health.

Kosobuski, A. W., King-Siert, R., Goodsky, T., Rodgers, J., and Thompson, H. (2020) *Minwanjige, Mino-bimaadizi (eat well, live well)*. Duluth, MN: University of Minnesota Medical School.
